# Potassium Humate and *Bacillus aryabhattai* Modulate Osmotic and Oxidative Homeostasis and Promote Cowpea Yield Under Drought

**DOI:** 10.3390/plants15172629

**Published:** 2026-08-28

**Authors:** Eulália Margarethe da Costa Melo, Agda Malany Forte de Oliveira, Semako Ibrahim Bonou, Priscylla Marques de Oliveira Viana, Igor Eneas Cavalcante, Guilherme Félix Dias, Ana Clara da Silva Dantas, Túlio William da Silva Gonçalves, Emmanuelly Silva Dias de Farias, Jessica Agra Guimarães, Liziane Maria de Lima, Alberto Soares de Melo

**Affiliations:** 1 Postgraduate Program in Agricultural Sciences, Paraiba State University, Campina Grande 58429-500, PB, Brazil; eulaliamelo91@gmail.com (E.M.d.C.M.); agda.forte@visitante.uepb.edu.br (A.M.F.d.O.); bonouibrahim@gmail.com (S.I.B.); pri.viana.marques@gmail.com (P.M.d.O.V.); guilhermefelix038@gmail.com (G.F.D.); jessicaagra513@gmail.com (J.A.G.); 2 Postgraduate Program in Agronomy, Federal University of Paraiba, Areia 58397-000, PB, Brazil; cavalcanteigor028@gmail.com; 3 Department of Biology, Paraiba State University, Campina Grande 58429-500, PB, Brazil; clara.acsd13@gmail.com (A.C.d.S.D.); tulio.goncalves@aluno.uepb.edu.br (T.W.d.S.G.); emmanuelly.farias@aluno.uepb.edu.br (E.S.D.d.F.); 4 Brazilian Agricultural Research Corporation, EMBRAPA Cotton, Campina Grande 58428-095, PB, Brazil; liziane.lima@embrapa.br

**Keywords:** *Vigna unguiculata*, biostimulants, drought stress, plant growth-promoting rhizobacteria (PGPR), humic substances, antioxidant system

## Abstract

Global climate change has intensified prolonged droughts, making the development of sustainable agricultural technologies essential. Although potassium humate (KH) and *Bacillus aryabhattai* (BA) are recognized individually as effective biostimulants, their joint application represents an innovative, unexplored complementary strategy to mitigate drought stress in cowpea (*Vigna unguiculata* L. Walp.). This study aimed to evaluate the effects of these bio-inputs on mitigating water stress in the cowpea (*Vigna unguiculata* L. Walp.) cultivar ‘BRS Verdejante’. The experiment was conducted under greenhouse conditions using a completely randomized design in a 2 × 8 factorial arrangement, with two water regimes (100% and 50% crop evapotranspiration—ETc) and eight biostimulant combinations of KH and BA. While BA alone (T4) intensified osmotic adjustment via proline accumulation and APX activation, the combined treatment T5 (10 mg kg^−1^ soil KH + 4 mL kg^−1^ seed BA containing 1 × 10^8^ CFU mL^−1^) achieved the best overall performance under drought (50% ETc). T5 effectively enhanced superoxide dismutase activity, preserved leaf relative water content, and reduced cell membrane electrolyte leakage by 42.6% compared to untreated stressed plants. These physiological adaptations protected reproductive organs, resulting in a 51.6% increase in pod number per plant under drought. The strategic co-application of low-dose potassium humate (10 mg kg^−1^ soil) and *B. aryabhattai* (4 mL kg^−1^ seed) effectively regulates osmotic and oxidative homeostasis, maintaining pod formation (+51.6%) and securing cowpea grain yield per plant under drought stress, thus serving as a promising strategy to mitigate drought stress in cowpea under controlled greenhouse conditions.

## 1. Introduction

Global climate change has been widely documented in recent decades, with the recent 2023–2025 triennium standing out as the warmest in 176 years of observational records [1]. The significant increase in temperatures, combined with irregular precipitation patterns, has intensified extreme climatic events, such as prolonged droughts [2,3]. These events, coupled with exponential population growth, have exacerbated the challenges facing global agriculture, limiting development and crop yields and directly threatening global food security [2,3].

This scenario is particularly critical in arid and semi-arid regions, which, despite being naturally characterized by irregular rainfall, high solar radiation, and elevated air temperatures, are highly vulnerable to climate change [4,5]. In the Brazilian semi-arid region, drought stress caused by the scarcity and poor distribution of rainfall constitutes the main abiotic factor limiting agricultural production [6]. Water deficit negatively affects multiple stages of plant development, highlighting the urgent need to identify and manage crops strategically adapted to restrictive environments [7].

Among the species cultivated in the semi-arid region, cowpea (*Vigna unguiculata* (L.) Walp.) stands out as a high-protein legume of immense socioeconomic importance for regional family farming, generating employment and income [8,9,10]. Despite its rusticity and ability to grow in low-fertility soils, cowpea productivity remains severely reduced by water stress under semi-arid conditions [4,11]. Under water restriction, the species triggers physiological and biochemical responses that include early stomatal closure [12], modulation of primary and secondary metabolite pathways [13], and the accumulation of compatible osmolytes for osmotic adjustment [14]. However, these survival mechanisms frequently occur at the expense of plant growth and grain yield [13,14].

In addition to climate change, the massive and indiscriminate use of synthetic agrochemicals in conventional agriculture to mitigate biotic challenges and ensure yield has resulted in ecosystem degradation through leaching and chemical contamination, rendering these production systems unsustainable in the long term [15,16,17]. This issue impels the search for ecologically viable technologies that mitigate abiotic stresses and maximize water and nutrient use efficiency while maintaining farm profitability [7,16,18]. In this context, bioinputs emerge as essential technological tools for transitioning toward sustainable agriculture [19,20], offering a favorable cost–benefit ratio and substantially reducing environmental impacts compared to synthetic inputs [21,22,23].

Among potential bioinputs, potassium humate (KH), a biostimulant rich in humic substances, and the plant growth-promoting rhizobacterium (PGPR) *B. aryabhattai* (BA) represent promising complementary strategies to mitigate drought [24,25]. Rather than operating through isolated pathways, these two bioinputs can trigger distinct yet complementary mechanisms within the rhizosphere and plant tissues. Soil-applied KH improves soil aggregation, cation exchange capacity, and water retention capacity, while enhancing root hydraulic conductance and nutrient availability [26,27,28]. This optimized physicochemical microenvironment fosters an ideal rhizosphere niche that promotes root colonization, survival, and biological activity of inoculated *B. aryabhattai*. In turn, *B. aryabhattai* secretes exopolysaccharides that form protective biofilms around roots, produces growth-regulating phytohormones, and synthesizes signaling molecules that stimulate the host plant’s antioxidant enzyme machinery and osmolyte accumulation [25,29,30]. Together, the physical and chemical properties of humic macromolecules, combined with bacterial inoculation, provide a system that preserves cell turgor, mitigates oxidative damage, and maintains plant metabolic activity during severe water restriction.

Although the individual biostimulatory effects of potassium humate (KH) and plant growth-promoting rhizobacteria (PGPR) on crop tolerance to abiotic stress have been documented in species such as maize, soybean, wheat, common beans, and yellow passion fruit [24,27,30,31,32,33,34], a critical research gap remains regarding their joint application under drought conditions. Specifically, in cowpea (*V. unguiculata* (L.) Walp.), an essential crop for food security in semi-arid regions, the interactive pathways by which humic substances and *B. aryabhattai* (BA) modulate osmotic adjustment, enzymatic antioxidant defenses, and cell membrane integrity under water restriction have not been systematically investigated. It remains unclear whether their co-application provides superior physiological protection compared with individual applications, and how these biochemical adaptations translate directly into grain yield protection under severe water stress.

Considering the socioeconomic importance of cowpea in semi-arid regions and the severe metabolic disruptions caused by drought, this study was based on the hypothesis that the co-application of potassium humate (KH) and *B. aryabhattai* (BA) provides superior enhanced physiological and biochemical protection compared with individual applications under water restriction. Therefore, the objective of this study was to evaluate the physiological, biochemical, and agronomic responses of cowpea (cv. ‘BRS Verdejante’) to various combinations of potassium humate (KH) and *B. aryabhattai* (BA) and to identify the most effective formulation for drought mitigation.

## 2. Results

### 2.1. Water Status Indicators and Membrane Damage

The interaction between biostimulant combinations and irrigation regimes significantly affected relative water content (RWC) (*p* ≤ 0.05), electrolyte leakage (EL), and lipid peroxidation (MDA) (*p* ≤ 0.01) (Table 1).

**Figure 1:**
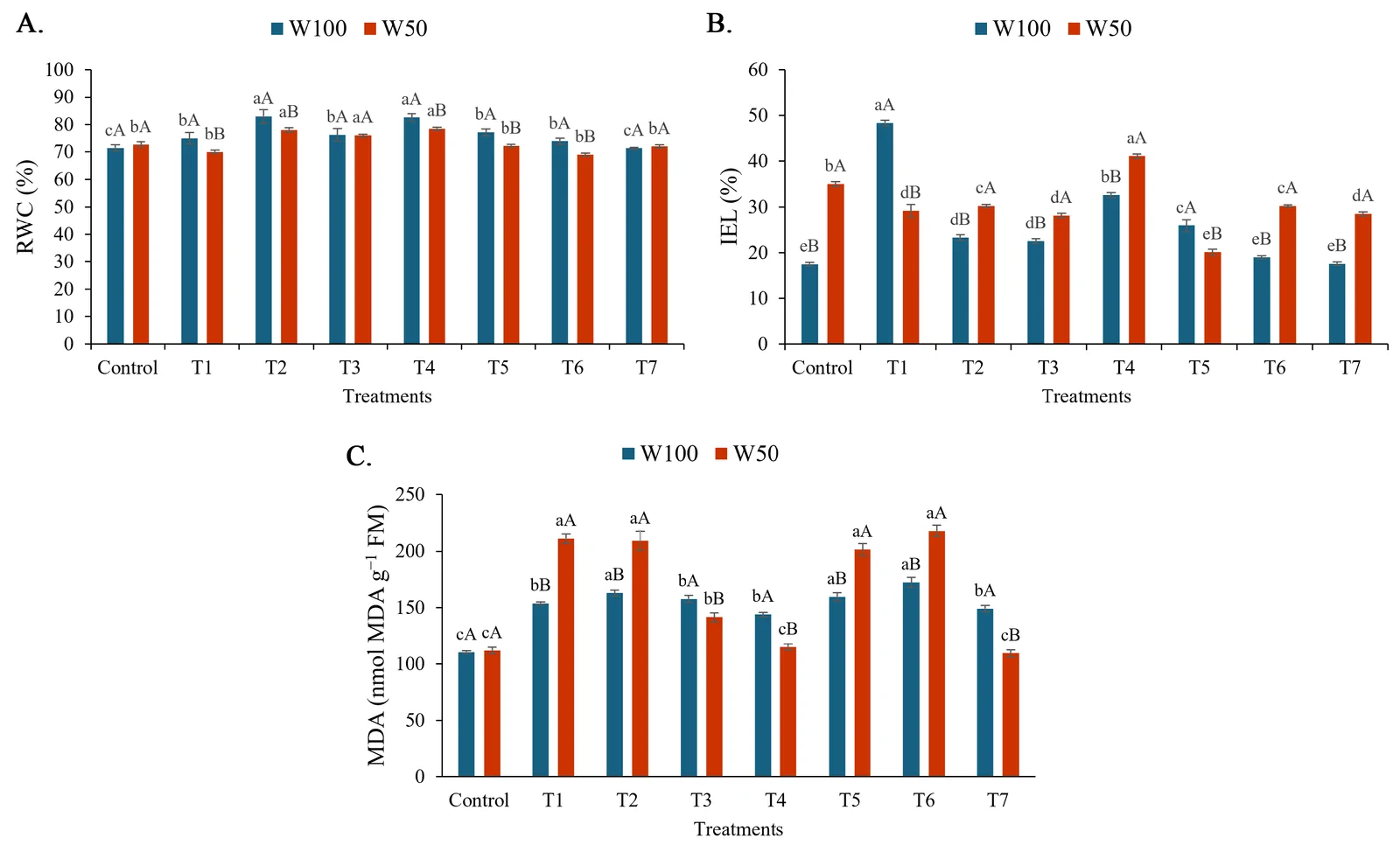
Relative water content (RWC) (**A**), electrolyte leakage (EL) (**B**), and lipid peroxidation (MDA) (**C**) in cowpea plants (*Vigna unguiculata* cv. ‘BRS Verdejante’) subjected to eight different combinations of potassium humate (KH) and *Bacillus aryabhattai* (BA) (control—without biostimulant application; T1—10 mg kg^−1^ soil KH; T2—15 mg kg^−1^ soil KH; T3—20 mg kg^−1^ soil KH; T4—4 mL kg^−1^ seeds BA; T5—10 mg kg^−1^ soil KH + 4 mL kg^−1^ seeds BA; T6—15 mg kg^−1^ soil KH + 4 mL kg^−1^ seeds BA; T7—20 mg kg^−1^ soil KH + 4 mL kg^−1^ seeds BA) under two irrigation regimes (100%—W100—and 50%—W50—ETc). Uppercase letters compare irrigation regimes within each biostimulant combination (Student’s *t*-test, *p* ≤ 0.05), and lowercase letters compare biostimulant combinations within each irrigation regime (Scott–Knott test, *p* ≤ 0.05).

Under full irrigation (W100), plants under T2 (15 mg kg^−1^ soil KH) and T4 (4 mL kg^−1^ BA) exhibited the highest relative water content (RWC), showing increases of 17.1% and 17.8% compared to control plants. Under drought stress (W50), plants under T2, T3, and T4 maintained higher leaf hydration, with RWC values of 9.2%, 4.6%, and 8.1% above untreated stressed controls (Figure 1A).

Regarding membrane integrity under drought conditions, all biostimulant applications reduced electrolyte leakage (EL) compared with non-conditioned stressed plants. Soil conditioning with potassium humate attenuated membrane damage under water deficit, with the greatest protection achieved by combining low humate doses with bacteria. Specifically, plants under the combined treatment T5 (10 mg kg^−1^ soil KH + 4 mL kg^−1^ BA, delivering 1 × 10^8^ CFU mL^−1^) showed the greatest membrane protection, reducing EL by 42.6% relative to the drought control (Figure 1B). For lipid peroxidation, plants under T4 and T7 maintained the lowest MDA contents under water restriction (114.9 and 109.4 nmol g^−1^ FM), remaining statistically similar to well-watered controls (Figure 1C).

### 2.2. Photosynthetic Pigments

The content of photosynthetic pigments, chlorophyll *a* (Chl *a*), chlorophyll *b* (Chl *b*) (*p* ≤ 0.05), total chlorophyll (Chl *t*), total carotenoids (CAR), and total anthocyanins (ANT) (*p* ≤ 0.01), was significantly affected by the interaction between biostimulant combinations and irrigation regimes (Table 2).

**Figure 2:**
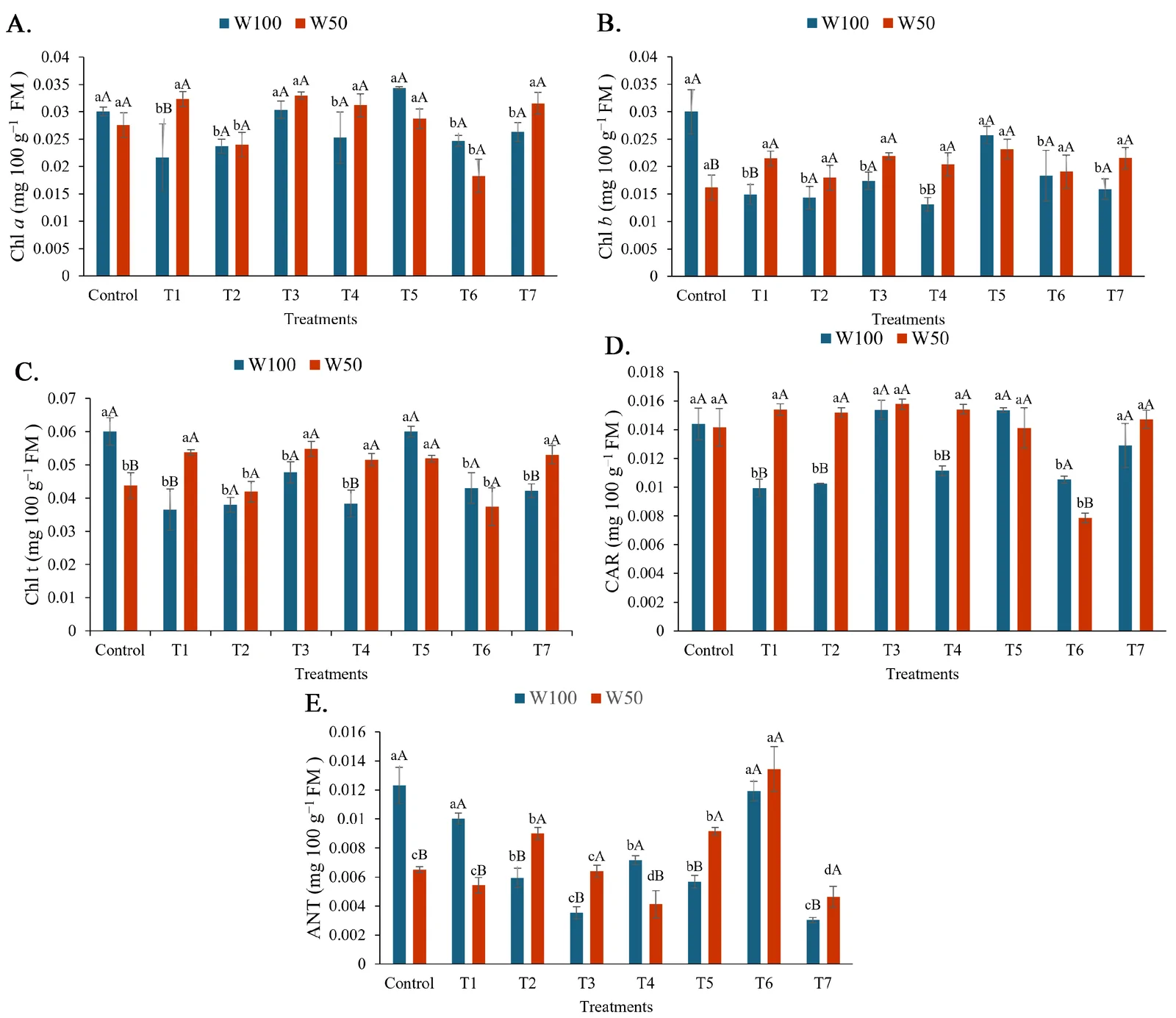
Chlorophyll *a* (Chl *a*) (**A**), Chlorophyll *b* (Chl *b*) (**B**), Total Chlorophyll (Chl *t*) (**C**), Carotenoids (CAR) (**D**), and Anthocyanins (ANT) (**E**) in cowpea plants (*Vigna unguiculata* cv. ‘BRS Verdejante’) subjected to different combinations of potassium humate (KH) and *Bacillus aryabhattai* (BA) (control—without biostimulant application; T1—10 mg kg^−1^ soil KH; T2—15 mg kg^−1^ soil KH; T3—20 mg kg^−1^ soil KH; T4—4 mL kg^−1^ seeds BA; T5—10 mg kg^−1^ soil KH + 4 mL kg^−1^ seeds BA; T6—15 mg kg^−1^ soil KH + 4 mL kg^−1^ seeds BA; T7—20 mg kg^−1^ soil KH + 4 mL kg^−1^ seeds BA) under two irrigation regimes (100%—W100—and 50%—W50—ETc). Uppercase letters compare irrigation regimes within each biostimulant combination (Student’s *t*-test, *p* ≤ 0.05), and lowercase letters compare biostimulant combinations within each irrigation regime (Scott–Knott test, *p* ≤ 0.05).

Under the W100 regime, the highest chlorophyll *a* contents were observed in plants under treatments T5 and T3, which were not statistically different from the control. In contrast, the other treatments showed reductions, with the lowest in T1, at 27.9% relative to the control. Under drought stress, only plants under T2 and T6 exhibited significantly lower Chl *a* contents than the control, with reductions of 13% and 33.6%, respectively. The remaining treatments maintained higher values and did not differ statistically from each other (Figure 2A). The effect of irrigation regime was significant only for plants under T1, where drought stress (W50) increased Chl *a* content by 49.4% compared to W100 (Figure 2A).

Under full irrigation (W100), the highest chlorophyll *b* content was observed in plants under T5, which remained statistically comparable to the control. The other treatments resulted in lower values and showed no significant differences among themselves. Under water restriction (W50), all biostimulant treatments maintained plant Chl *b* content, with no statistically significant differences among them (Figure 2B). Regarding irrigation regimes, plants under water deficit associated with T1 and T4 exhibited increases of 43.7% and 55.5% in chlorophyll *b* compared to well-watered plants. In contrast, control plants showed a 46% lower chlorophyll content under limited irrigation (Figure 2B).

Under well-watered conditions (W100), the highest total chlorophyll content was observed in plants under T5, which was not significantly different from the control (Figure 2C). The remaining treatments reduced plant Chl *t* under W100. Conversely, under drought stress (W50), treatments T1, T3, T4, T5, and T7 increased plant Chl *t* by 22.9%, 25.5%, 17.8%, 18.6%, and 21.3%, respectively, compared to control plants (Figure 2C). Unconditioned control plants under drought (W50) experienced a 27% reduction in Chl *t* compared to well-watered controls. In contrast, plants treated with T1, T4, and T7 under W50 showed increases of 47%, 34.3%, and 25.8% relative to the same treatments under W100, indicating that these biostimulants preserved photosynthetic pigments under drought. Most treatments raised total plant chlorophyll under low water supply, except for the medium humate dose used alone or with *B. aryabhattai*.

Regarding total carotenoids, plants treated with T1, T2, T4, and T6 under W100 showed reductions in carotenoid synthesis of 30.9%, 28.7%, 22.5%, and 26.8%, respectively, compared to control plants. Under water deficit, only T6 caused a significant reduction in carotenoid content (44.5%), while the other treatments did not differ from the control (Figure 2D). Evaluating irrigation regimes within each treatment, T1, T2, and T4 under W50 increased plant carotenoid levels by 54.8%, 48%, and 38.1% compared to well-watered conditions, whereas T6 showed an inverse trend, with a 25.4% reduction under W50 (Figure 2D).

The lowest anthocyanin values in plants under W100 were observed in T3 and T7, representing reductions of 71% and 75%, respectively, compared to the control. Under drought stress, the highest value was observed in plants under T6. In contrast, the lowest values were observed in plants with T4 and T7, which were statistically equal and showed reductions of 36% and 28%, respectively, compared to control plants under W50 (Figure 2E). Except for plants under T6, all treatments showed significant differences in anthocyanin among irrigation regimes. The plants under control, T1, and T4 treatments presented higher values under W100 (47%, 45%, and 42% higher than W50), whereas T2, T3, T5, and T7 plants exhibited lower values under W100, representing increases of 51%, 81%, 61%, and 52% under W50 (Figure 2E).

### 2.3. Antioxidant Defense System Activity

The interaction between biostimulant combinations and irrigation regimes significantly influenced the activities of superoxide dismutase (SOD), catalase (CAT), and ascorbate peroxidase (APX) (*p* ≤ 0.01) (Table 3).

**Figure 3:**
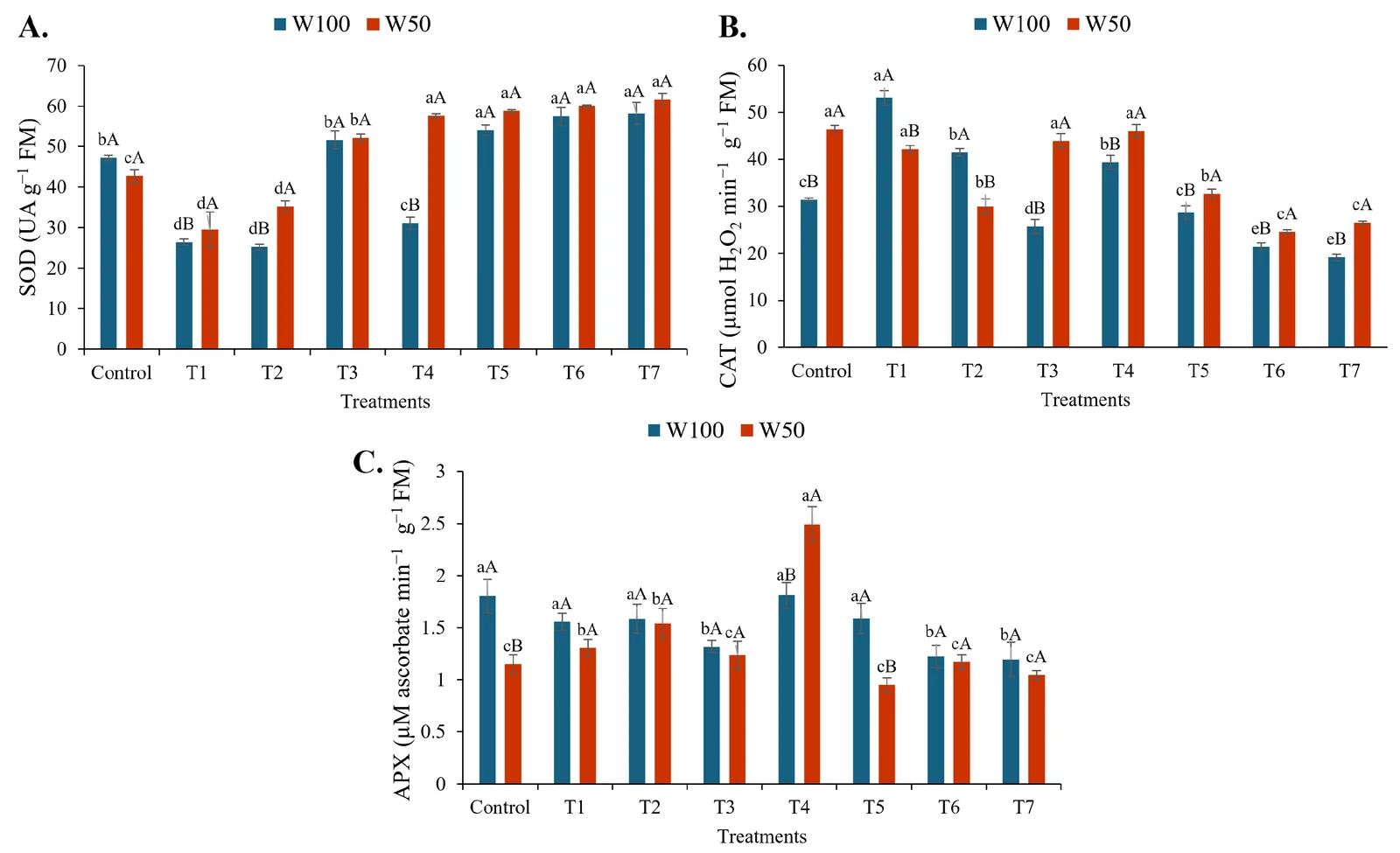
Superoxide dismutase (SOD) (**A**), catalase (CAT) (**B**), and ascorbate peroxidase (APX) (**C**) activities in cowpea plants (*Vigna unguiculata* cv. ‘BRS Verdejante’) subjected to different combinations of potassium humate (KH) and *Bacillus aryabhattai* (BA) (control—without biostimulant application; T1—10 mg kg^−1^ soil KH; T2—15 mg kg^−1^ soil KH; T3—20 mg kg^−1^ soil KH; T4—4 mL kg^−1^ seeds BA; T5—10 mg kg^−1^ soil KH + 4 mL kg^−1^ seeds BA; T6—15 mg kg^−1^ soil KH + 4 mL kg^−1^ seeds BA; T7—20 mg kg^−1^ soil KH + 4 mL kg^−1^ seeds BA) under two irrigation regimes (100%—W100—and 50%—W50—ETc). Uppercase letters compare irrigation regimes within each biostimulant combination (Student’s *t*-test, *p* ≤ 0.05), and lowercase letters compare biostimulant combinations within each irrigation regime (Scott–Knott test, *p* ≤ 0.05).

Superoxide dismutase (SOD) activity was maximized in plants under combined treatments (T5, T6, and T7), with activity remaining high (54.0 to 61.0 UA g^−1^ FM) regardless of water availability. The highest humate dose increased SOD activity under low water supply (W50). Adding bacteria made this effect even stronger. Notably, inoculation with only *B. aryabhattai* (T4) led to an 85.5% increase in SOD activity under W50 compared with full irrigation (Figure 3A). This boost did not depend on the addition of humates, indicating that bacterial inoculation alone was the primary driver of SOD upregulation under water restriction.

Under drought (W50), plants in the T1, T3, and T4 treatments exhibited the highest catalase activities, which were not statistically different from those of control plants. Under W100, T1, T2, and T4 plants resulted in higher CAT activity than the control, corresponding to increases of 68.9%, 32.1%, and 25.3%, respectively. Under both water conditions, T6 and T7 reduced plant CAT activity by 31.8% and 39% (W100), and by 46.9% and 42.8% (W50), respectively, relative to their respective controls. Only plants under T1 and T2 showed higher CAT activity under W100 than under W50 (20.6% and 27.8% higher, respectively) (Figure 3B).

Under full irrigation (W100), treatments T3, T6, and T7 reduced plant ascorbate peroxidase activity by 29%, 32%, and 33%, respectively, compared to the control, whereas the other treatments showed no significant differences. Under drought stress (W50), the plants under T4 showed a 116% increase in APX activity relative to the control, followed by T1 (13.7%) and T2 (34.1%), which were not statistically different from each other. The lowest APX activities under drought were observed in the plants under control, T3, T5, T6, and T7, which were statistically equal. Significant differences between irrigation regimes occurred only in the control (a 36.4% reduction under W50), T4 (a 37.5% increase under W50), and T5 (a 40.2% reduction under W50) (Figure 3C).

### 2.4. Osmotic Adjustment Mechanism

The interaction between biostimulant combinations and irrigation regimes significantly influenced free proline (PRO) and total soluble sugars (TSS) (*p* ≤ 0.05), as well as total soluble proteins (TSP) (*p* ≤ 0.01) (Table 4).

**Figure 4:**
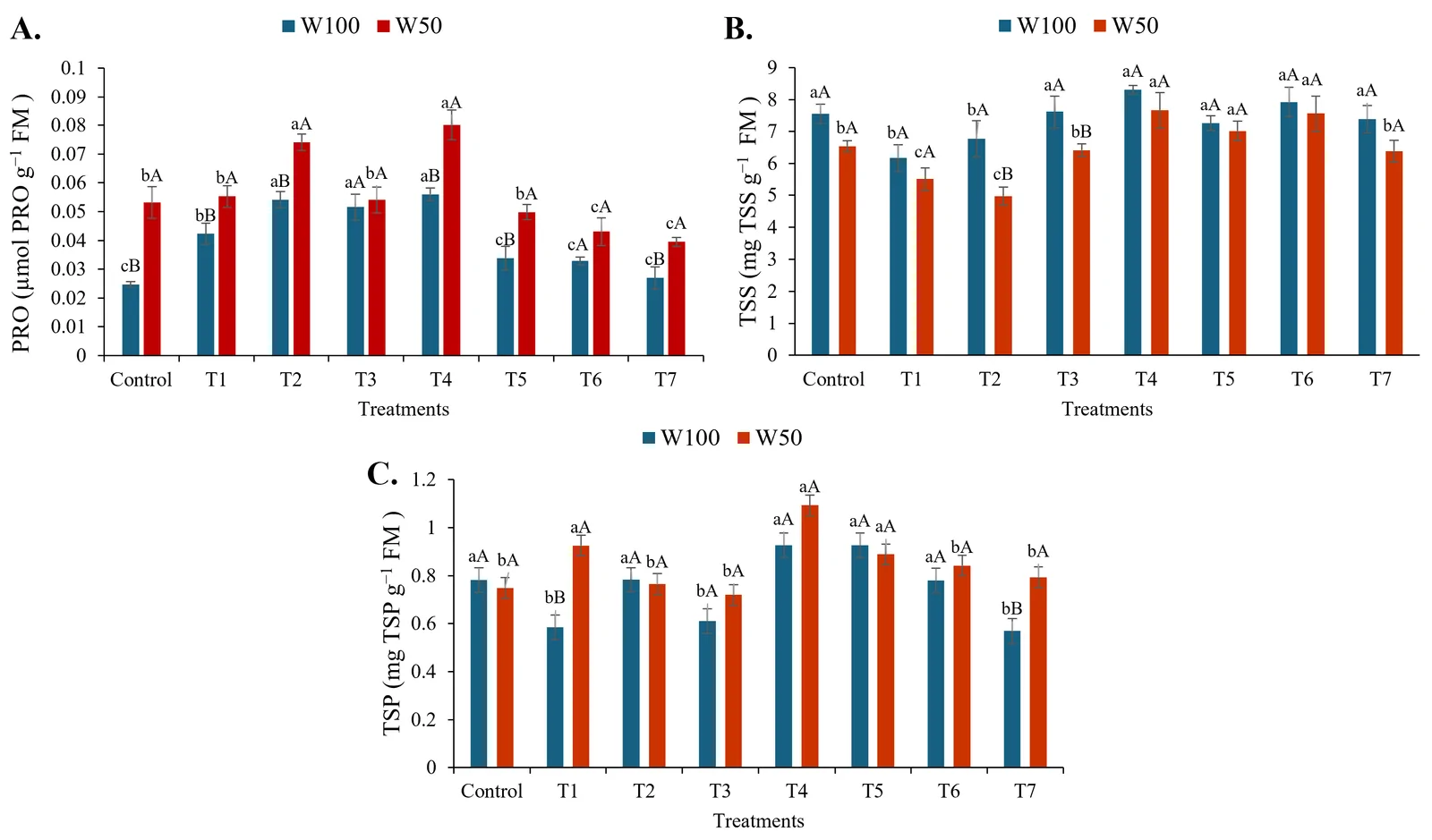
Free proline (PRO) (**A**), Total Soluble Sugars (TSS) (**B**), and Total Soluble Proteins (TSP) (**C**) in cowpea plants (*Vigna unguiculata* cv. ‘BRS Verdejante’) subjected to different combinations of potassium humate (KH) and *Bacillus aryabhattai* (BA) (control—without biostimulant application; T1—10 mg kg^−1^ soil KH; T2—15 mg kg^−1^ soil KH; T3—20 mg kg^−1^ soil KH; T4—4 mL kg^−1^ seeds BA; T5—10 mg kg^−1^ soil KH + 4 mL kg^−1^ seeds BA; T6—15 mg kg^−1^ soil KH + 4 mL kg^−1^ seeds BA; T7—20 mg kg^−1^ soil KH + 4 mL kg^−1^ seeds BA) under two irrigation regimes (100%—W100—and 50%—W50—ETc). Uppercase letters compare irrigation regimes within each biostimulant combination (Student’s *t*-test, *p* ≤ 0.05), and lowercase letters compare biostimulant combinations within each irrigation regime (Scott–Knott test, *p* ≤ 0.05).

Higher free proline content was observed in plants under drought stress (W50) compared to well-watered conditions across all treatments, except in T3, which showed no significant differences between regimes. Under full irrigation (W100), the highest proline contents were observed in plants under T2, T3, and T4, which were not statistically different and represented increases of 119%, 108%, and 126%, respectively, compared to the control. The lowest values were recorded in the control, T5, T6, and T7 plants. Under drought (W50), the highest PRO contents were observed in plants under T2 and T4, representing increases of 39% and 50%, respectively, relative to the control. In contrast, the lowest contents were observed in plants subjected to T6 and T7, resulting in reductions of 18.9% and 25.6% compared to the control (Figure 4A).

Under both irrigation regimes, plants in T1 and T2 exhibited reduced total soluble sugar (TSS) accumulation compared to their respective controls (18.4% and 10.3% lower under W100; 15.7% and 23.9% lower under W50). Plants under other treatments did not differ in TSS when sufficient water was supplied. However, under drought stress (W50), plants under T4, T5, and T6 recorded the highest TSS values, increasing by 17.2%, 7.2%, and 15.6%, respectively, compared to control plants (Figure 4B). Significant differences between irrigation regimes were detected only in plants under T2 and T3, where drought stress reduced plant biomass by 26.5% and 15.7%, respectively, relative to well-watered conditions (Figure 4B).

Regarding total soluble proteins (TSP) under W100, plants under T1, T3, and T7 exhibited the lowest TSP content, with reductions of 25.2%, 21.7%, and 27.1%, respectively, relative to the control. Under drought stress (W50), the highest TSP contents were observed in plants under T1, T4, and T5, representing increases of 23.5%, 45.9%, and 18.6%, respectively, compared to control plants (Figure 4C). Bacterial treatments under drought conditions effectively increased total soluble sugars (TSS). At the same time, low-dose humate and bacteria, applied alone and in combination, increased total soluble proteins (TSP) relative to controls, thereby enabling plant osmotic regulation.

### 2.5. Plant Growth

The interaction between irrigation regimes and biostimulant treatments significantly influenced vegetative growth parameters. Significant differences were detected for plant height (PH), stem diameter (SD), total leaf area (TLA), and shoot dry mass (SDM) (*p* ≤ 0.01) (Table 5).

**Figure 5:**
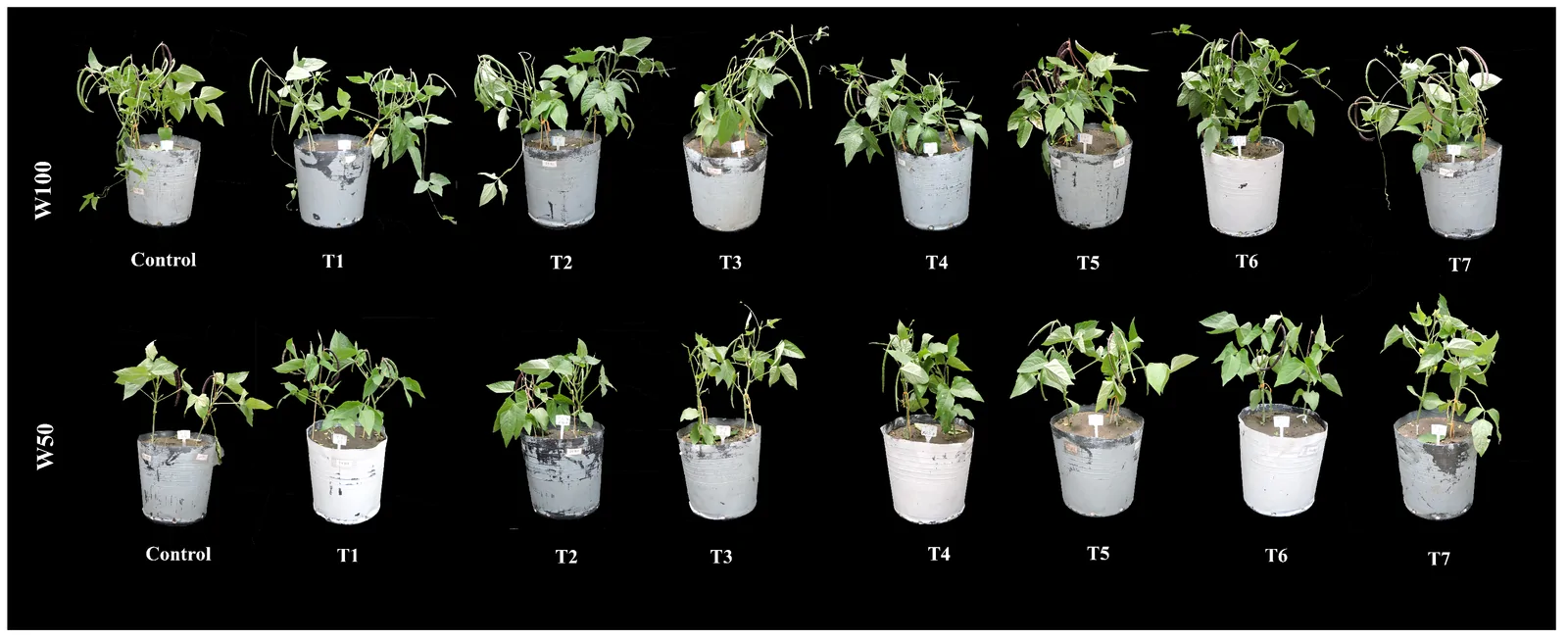
Visual appearance and vegetative growth of cowpea plants (*Vigna unguiculata* cv. ‘BRS Verdejante’) subjected to different combinations of potassium humate (KH) and *Bacillus aryabhattai* (BA) (control—without biostimulant application; T1—10 mg kg^−1^ soil KH; T2—15 mg kg^−1^ soil KH; T3—20 mg kg^−1^ soil KH; T4—4 mL kg^−1^ seeds BA; T5—10 mg kg^−1^ soil KH + 4 mL kg^−1^ seeds BA; T6—15 mg kg^−1^ soil KH + 4 mL kg^−1^ seeds BA; T7—20 mg kg^−1^ soil KH + 4 mL kg^−1^ seeds BA) under two irrigation regimes (100%—W100—and 50%—W50—ETc).

**Figure 6:**
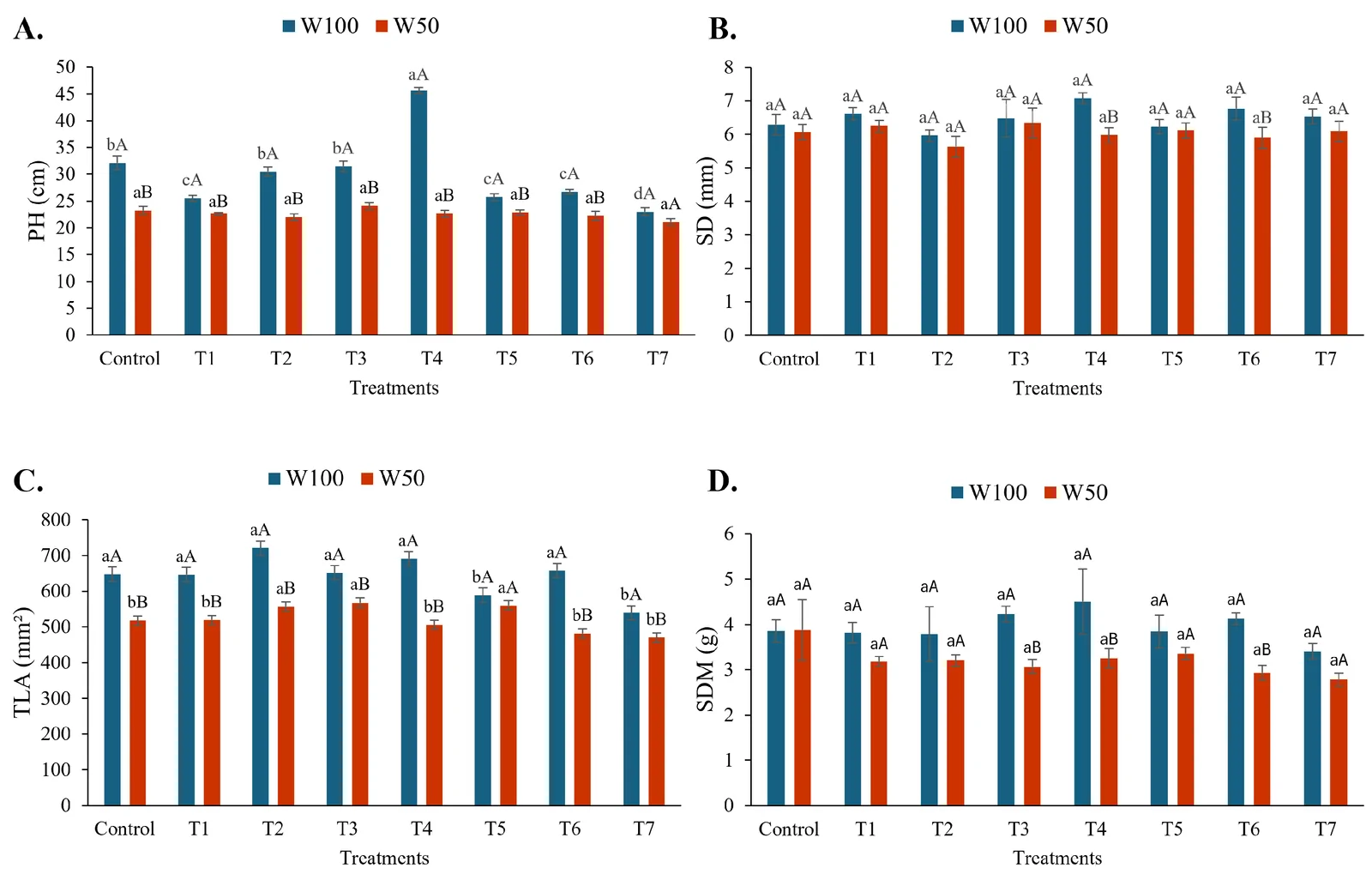
Plant Height (PH) (**A**), Stem Diameter (SD) (**B**), Total Leaf Area (TLA) (**C**), and Shoot Dry Mass (SDM) (**D**) in cowpea plants (*Vigna unguiculata* cv. ‘BRS Verdejante’) subjected to different combinations of potassium humate (KH) and *Bacillus aryabhattai* (BA) (control—without biostimulant application; T1—10 mg kg^−1^ soil KH; T2—15 mg kg^−1^ soil KH; T3—20 mg kg^−1^ soil KH; T4—4 mL kg^−1^ seeds BA; T5—10 mg kg^−1^ soil KH + 4 mL kg^−1^ seeds BA; T6—15 mg kg^−1^ soil KH + 4 mL kg^−1^ seeds BA; T7—20 mg kg^−1^ soil KH + 4 mL kg^−1^ seeds BA) under two irrigation regimes (100%—W100—and 50%—W50—ETc). Uppercase letters compare irrigation regimes within each biostimulant combination (Student’s *t*-test, *p* ≤ 0.05), and lowercase letters compare biostimulant combinations within each irrigation regime (Scott–Knott test, *p* ≤ 0.05).

The application of biostimulants was particularly effective under full irrigation (W100), with treatment T4 resulting in a 42% increase in plant height compared to the control. Under drought stress (W50), no statistically significant differences in PH were observed among biostimulant treatments. Regarding irrigation regimes, well-watered plants (W100) were taller than drought-stressed plants (W50) across all treatments, except for plants under T7, where W50 did not differ statistically from W100 (Figure 5 and Figure 6A).

Stem diameter (SD) showed stability across the experimental variables. No significant differences were observed among biostimulant applications within either W100 or W50 regimes. However, comparing water regimes, plants in treatments T4 and T6 under W50 resulted in SD reductions of 15% and 12%, respectively, relative to their well-watered counterparts (Figure 5 and Figure 6B).

The highest total leaf area (TLA) values were recorded in plants under well-watered conditions (W100) compared to drought stress (W50), except for T5 plants, which showed no significant difference between regimes. Under drought stress (W50), plants treated with T2, T3, and T5 exhibited increases of 7.7%, 9.6%, and 8.2% in TLA, respectively, compared to control plants under the same regime. Under well-watered conditions, plants in the T5 and T7 treatments showed TLA reductions of 9% and 16%, respectively, compared to the control (Figure 5 and Figure 6C).

Shoot dry mass (SDM) showed no statistical differences among biostimulant treatments within W100 or W50 regimes. Between water regimes, significantly higher shoot dry mass (SDM) values were observed in plants in T3, T4, and T6 under W100 than under W50, indicating drought-induced SDM reductions of 27.5%, 27.8%, and 28.8%, respectively (Figure 5 and Figure 6D).

### 2.6. Agronomic Yield Components

Grain yield per plant (GY), number of pods per plant (NP) (*p* ≤ 0.05), and 100-grain weight (HGW) (*p* ≤ 0.01) were significantly affected by the interaction between biostimulant combinations and irrigation regimes (Table 6).

**Figure 7:**
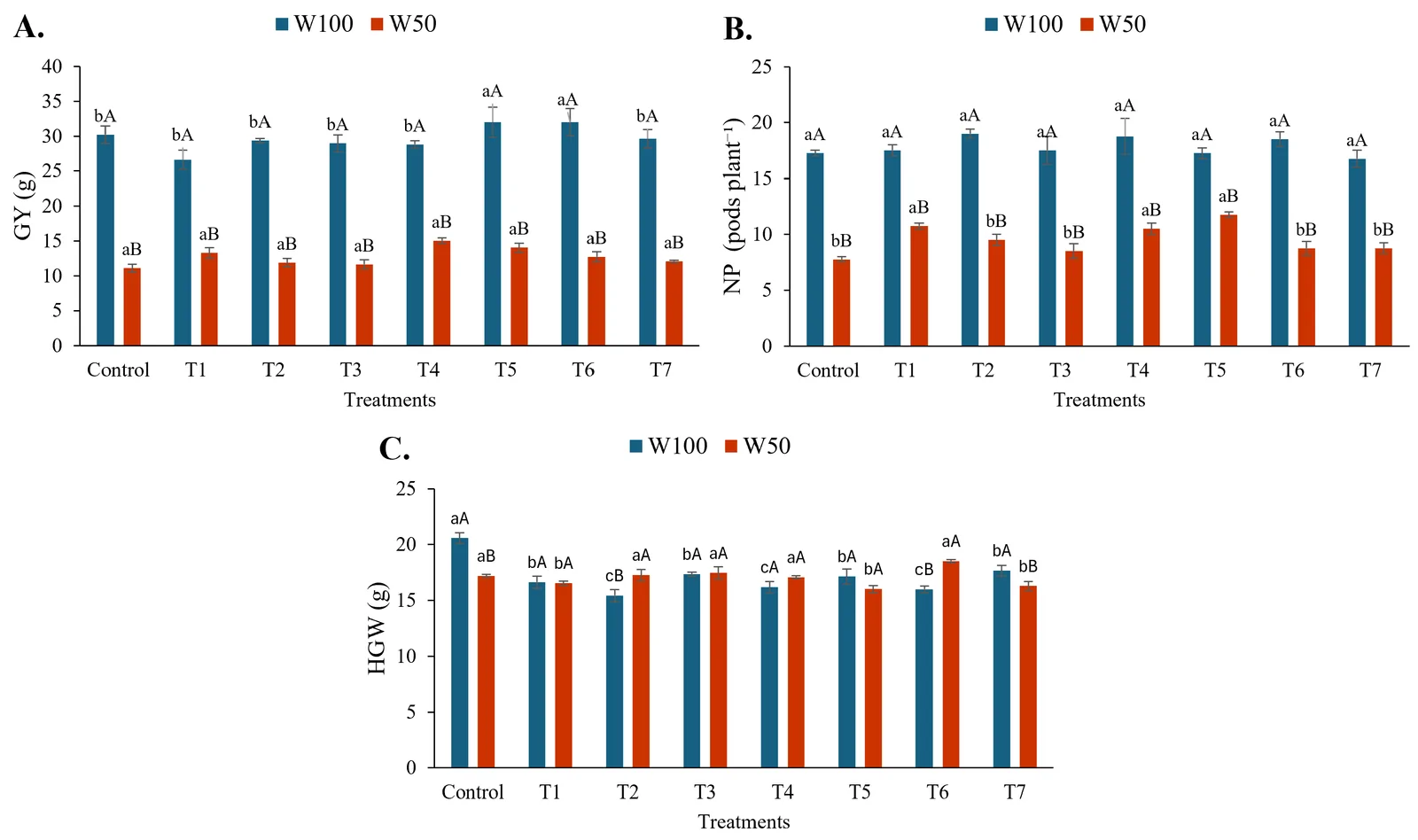
Grain yield per plant (GY) (**A**), number of pods per plant (NP) (**B**), and 100-grain weight (HGW) (**C**) in cowpea plants (*Vigna unguiculata* cv. ‘BRS Verdejante’) subjected to different combinations of potassium humate (KH) and *Bacillus aryabhattai* (BA) (control—without biostimulant application; T1—10 mg kg^−1^ soil KH; T2—15 mg kg^−1^ soil KH; T3—20 mg kg^−1^ soil KH; T4—4 mL kg^−1^ seeds BA; T5—10 mg kg^−1^ soil KH + 4 mL kg^−1^ seeds BA; T6—15 mg kg^−1^ soil KH + 4 mL kg^−1^ seeds BA; T7—20 mg kg^−1^ soil KH + 4 mL kg^−1^ seeds BA) under two irrigation regimes (100%—W100—and 50%—W50—ETc). Uppercase letters compare irrigation regimes within each biostimulant combination (Student’s *t*-test, *p* ≤ 0.05), and lowercase letters compare biostimulant combinations within each irrigation regime (Scott–Knott test, *p* ≤ 0.05).

The highest grain yield per plant was recorded under normal irrigation (W100) compared to drought stress (W50). Under W100, plants in treatments T5 and T6 increased grain yield per plant by 5.9% and 5.9%, respectively, compared to the control. Under drought stress (W50), no statistically significant differences in grain yield per plant were observed among treatments (Figure 7A).

Under drought (W50), the highest pod-per-plant values were recorded in plants receiving the T1, T4, and T5 treatments, representing increases of 22%, 35%, and 51%, respectively, relative to the control. No significant differences in the number of pods per plant were detected among treatments under normal irrigation. Across all treatments, higher pod numbers were maintained under W100 compared to W50 (Figure 7B).

Comparing water regimes for 100-grain weight (HGW), drought-stressed plants treated with T2 and T6 showed 100-grain weight increases of 11.7% and 15.8%, respectively, compared to well-watered plants. Combinations T1, T3, and T5 maintained similar 100-grain weight values across both water regimes. Under normal irrigation, the highest 100-grain weight was recorded in control plants, whereas biostimulant combinations yielded lower values. Under drought stress, the highest 100-grain weight values were obtained in plants in T6, T4, T3, T2, and the control (Figure 7C).

## 3. Discussion

Drought stress triggers severe physiological and biochemical disturbances in crops, primarily due to water restriction and increased production of reactive oxygen species (ROS) [11,13]. In cowpea (*Vigna unguiculata* (L.) Walp. cv. ‘BRS Verdejante’), the severe water deficit applied (W50) increased lipid peroxidation and damaged cellular membranes, as shown by higher electrolyte leakage and malondialdehyde (MDA) levels in untreated control plants (Figure 1). These metabolic damages directly reduced vegetative growth and grain yield per plant component (Figure 5, Figure 6 and Figure 7).

However, applying potassium humate (KH) to the soil and inoculating seeds with *B. aryabhattai* (BA), especially when combined, effectively altered cowpea plants’ responses to drought. Instead of using energy solely for vegetative biomass, these bioinputs redirected the plant’s metabolism toward osmotic adjustment and antioxidant defenses. This strategy helped maintain cellular balance and protected yield components under water restriction [24,27,35].

Maintaining cellular water *status* and membrane stability is essential for drought tolerance. In the present study, soil conditioning with potassium humate attenuated drought-induced electrolyte leakage, with the maximum protective effect achieved when a low dose of humate was co-applied with *B. aryabhattai* (T5). This response suggests that humate-mediated improvements in soil physical properties, coupled with bacterial physiological priming, stabilize cell membranes against dehydration-induced oxidative damage. The preservation of leaf relative water content (RWC) in untreated control plants under water restriction (W50) may be associated with the drought avoidance mechanism of cowpea (*Vigna unguiculata*) [13,19]. When exposed to water deficits, cowpea rapidly closes stomata to limit transpirational water loss while prioritizing cellular hydration in existing leaves at the expense of leaf area expansion and biomass accumulation [13,26]. However, while untreated control plants maintained RWC at the expense of growth, they exhibited severe cell membrane damage (elevated EL and MDA). In contrast, biostimulant-treated plants, particularly those receiving *B. aryabhattai* alone (T4) or in combination with potassium humate (T5), maintained high RWC, along with enhanced cell membrane protection and active enzymatic antioxidant defense, thereby avoiding the oxidative penalties associated with dehydration.

When combined with *B. aryabhattai*, this protective response was intensified. *B. aryabhattai* colonizes the rhizosphere and secretes exopolysaccharides that help retain moisture around the root zone. Furthermore, the literature suggests that PGPR inoculation can induce host aquaporin expression and modify root architecture, potentially enhancing water uptake during drought periods [25,29,35]. Similar protective effects on tissue hydration and membrane integrity were reported by Alharbi et al. [24] in soybean (*Glycine max* (L.) Merr.) and Taha and Osman [36] in common bean (*Phaseolus vulgaris* L.) under water restriction.

Regarding lipid peroxidation, leaf MDA levels remained within a relatively stable physiological baseline in control plants under both water regimes, which aligns with cowpea’s rapid stomatal closure and drought-tolerance mechanisms that restrict membrane lipid breakdown [13,19]. Consequently, the cellular protection conferred by the biostimulant combinations, specifically T5, is primarily expressed through the preservation of membrane-selective permeability (reduced EL) (Figure 1C) and enhanced enzymatic ROS scavenging, thereby preventing structural degradation of leaf tissues under stress [25,29,37,38].

This membrane protection may be associated with PGPR-triggered physiological priming [25,35]. Previous studies report that *B. aryabhattai* can produce signaling molecules and phytohormones that stimulate plant secondary metabolism, which helps stabilize cellular membranes against oxidative degradation under stress [25,35,39]. Although responses to potassium humate alone can vary depending on the application route and crop species, such as foliar potassium humate reducing MDA in *Allium cepa* L. [40] or *Oryza sativa* L. under salt stress [41], our findings show that soil-applied KH requires adequate bacterial association to effectively minimize membrane damage under drought. This protective mechanism on cellular membranes aligns with findings reported by Alharbi et al. [24] in *Glycine max* and Palmeira et al. [34], where joint applications of *B. aryabhattai* with soil amendments enhanced membrane stability, reduced electrolyte leakage, and attenuated lipid peroxidation under water deficit compared to isolated treatments. This combined attenuation indicates that integrating humic substances with plant growth-promoting rhizobacteria can provide antioxidant protection and preserve membrane integrity under abiotic stress.

Preserving photosynthetic pigments during drought is another protection mechanism activated by these bioinputs. While untreated control plants showed a sharp decrease in chlorophyll *b* and total chlorophylls under stress (Figure 2B,C), plants treated with the bioinputs maintained or even increased their pigment levels. This preservation happens for two main reasons: first, humic substances stimulate enzymes involved in chlorophyll synthesis; second, enhanced antioxidant activity protects chloroplast membranes from photo-oxidative damage [23,24].

Additionally, the increase in protective accessory pigments, such as carotenoids and anthocyanins, in specific combinations (Figure 2D,E) demonstrates an active energy-dissipation strategy. Carotenoids safely absorb excess light energy, preventing damage to reaction centers under stress conditions [13,37]. These physiological benefits align with findings from distinct plant species, in which seed priming or soil conditioning with *B. aryabhattai* and potassium humate effectively sustained photosynthetic pigment stability under stress, as reported for *Solanum lycopersicum* [39], *Arabidopsis thaliana* [23], and *Glycine max* [24].

To handle oxidative stress, plants activate an enzymatic antioxidant system. Superoxide dismutase acts as the first line of defense, converting superoxide radicals (•O_2_^−^) into hydrogen peroxide H_2_O_2_ and oxygen [13,41]. Subsequently, enzymes such as catalase (CAT) and ascorbate peroxidase detoxify H_2_O_2_ into water [13,31,42]. In this study, treatments containing *B. aryabhattai* (either alone as T4 or combined with KH as T5, T6, and T7) significantly increased superoxide dismutase activity under drought conditions (Figure 3A). This bacterium secretes metabolic compounds that can act as physiological signals, priming the host plant’s enzymatic antioxidant machinery [29,35,41]. As documented in the literature, such microbial priming is frequently associated with upregulation of candidate antioxidant enzyme transcripts, potentially explaining the higher enzymatic activities observed in our assay [29,41]. However, downstream enzyme responses varied; applying BA alone (T4) coordinated the activation of SOD, CAT, and APX (Figure 3), creating an efficient system for neutralizing ROS.

In contrast, combined treatments with higher KH doses (T6 and T7) reduced catalase and ascorbate peroxidase activities despite maintaining high superoxide dismutase levels (Figure 3B,C). This response suggests that higher doses of potassium humate, combined with bacteria, might alter the kinetics of ROS detoxification, possibly via alternative non-enzymatic antioxidant pathways or non-enzymatic ROS scavengers, as reported in other species subjected to humic substance conditioning [24,43]. On the other hand, in treatments where enzymatic activity was less pronounced, cowpea plants appear to have prioritized alternative desiccation-protection mechanisms, as evidenced by high proline (PRO) accumulation under stress (Figure 4A). Similar patterns of SOD stability, accompanied by variations in CAT and APX and coordinated osmoprotection under water restriction in cowpea, were also reported by Alencar et al. [43] and Oliveira et al. [44], highlighting the species’ physiological flexibility in triggering metabolic adjustments to preserve cellular homeostasis under drought.

In addition to antioxidant defenses, osmotic adjustment plays a crucial role in maintaining turgor and physiological function during dehydration [42,45]. Free proline (PRO) accumulation was stimulated by single applications of KH (T2) and BA (T4) under drought stress (Figure 4A). Proline acts not only as a primary osmoticum but also quenches hydroxyl radicals, stabilizes subcellular macromolecules, and provides energy reserves for cellular recovery following rehydration [42,46]. Plant growth-promoting bacteria like *B. aryabhattai* stimulate proline biosynthesis by upregulating key enzymatic pathways in host tissues [39,46]. These findings align with previous studies demonstrating enhanced proline accumulation mediated by *B. aryabhattai* seed priming or soil potassium, or KH conditioning under stress, as observed in cowpea (*Vigna unguiculata* L. Walp.) [45,47], tomato (*Solanum lycopersicum*) [39], and common bean (*Phaseolus vulgaris* L.) [36].

Similarly, total soluble sugars and total soluble proteins were actively modulated by biostimulant combinations under water restriction (Figure 4B,C). Combined BA treatments with low-to-moderate KH doses (T4, T5, and T6) induced greater accumulation of TSS and TSP under drought conditions. This response reflects a coordinated strategy to maintain cellular osmotic potential and preserve soluble protein stability, potentially involving stress-responsive functional proteins that protect cellular structures during dehydration [24,42,48].

This preservation of total soluble proteins and sugars under water restriction reflects an osmoprotective strategy mediated by biostimulants. A similar mechanism was reported in *Glycine max* with the joint application of potassium humate and plant growth-promoting microbes, in which total soluble protein levels were elevated despite reduced proline and antioxidant enzyme activities [24]. Although elevated TSS and TSP concentrations are common under moderate water deficit [48], severe drought or excessively high biostimulant doses can sometimes trigger osmolyte depletion due to metabolic impairment or altered carbon allocation, as observed in *Daucus carota* [49] and *Arabidopsis thaliana* [23]. In cowpea, however, the strategic combination of KH and BA preserved osmolyte accumulation, ensuring osmoregulation under water-limited conditions.

This preservation of osmotic solutes, together with membrane protection and yield maintenance in treatments like T5, reflects a broader physiological trend observed in dual PGPR–biostimulant systems. Beyond the interaction between *B. aryabhattai* and potassium humate, similar complementary responses have been documented for distinct biostimulant combinations under drought stress. These include the co-application of *Azospirillum brasilense* with humic substances in *Phaseolus vulgaris* [33], *Pseudomonas fluorescens* combined with seaweed extracts (*Ascophyllum nodosum*) in *Glycine max* [24], and *Rhizobium* co-inoculation with fulvic acids under water restriction [36]. Across these diverse systems, organic biostimulants consistently act as soil conditioners that improve rhizosphere moisture retention and the stability of physical niches, thereby facilitating bacterial survival and root colonization. In return, the inoculated PGPR triggers an osmotic adjustment in the host plant. Our findings in cowpea demonstrate that the joint application of *B. aryabhattai* and potassium humate shows similar behavior.

Regarding vegetative development, the water deficit throughout the vegetative stage imposed a high cost on the balance between growth expansion and maintenance of cellular defense [50]. Plants allocated carbon reserves primarily toward osmotic adjustment and antioxidant protection rather than stem elongation, leaf expansion, or shoot dry mass accumulation (Figure 5 and Figure 6). These findings contrast with reports in which biostimulant application promoted vegetative growth under moderate or transient stress, including increased height, leaf area, and biomass in *Phaseolus vulgaris* [33], *Zea mays* [31], and *Allium cepa* [40].

It is noteworthy that higher doses of potassium humate (20 mg kg^−1^ soil; T3 and T7) did not result in superior physiological or agronomic performance. This response can be attributed to the nature of humic macromolecules in plants, where low to moderate concentrations stimulate root activity and nutrient uptake, whereas high concentrations may cause slight metabolic suppression or growth reductions [23,26,51]. Under severe water stress (W50), high doses of potassium humate can increase electrical conductivity and impose slight osmotic stress on root membranes [26,51]. Furthermore, high concentrations of humic macromolecules may excessively chelate essential micronutrient cations (such as Fe, Mn, and Zn) in the rhizosphere, temporarily restricting their bioavailability as necessary cofactors for downstream antioxidant enzymes, such as catalase and ascorbate peroxidase [24,49]. Consequently, the application of potassium humate at a low dose (10 mg kg^−1^ soil; T1 and T5) yielded the most significant results in this study.

This occurs because, under severe or prolonged water restriction, the primary physiological drive shifts toward survival. Under severe water restriction, osmoregulation and enzymatic antioxidant machinery function primarily as protective mechanisms to maintain cellular homeostasis and prevent irreversible tissue desiccation. However, the biosynthesis of osmotic solutes (proline, soluble proteins) and the sustained turnover of antioxidant enzymes impose a high energetic cost, diverting photoassimilates away from vegetative biomass expansion and yield accumulation [50,51]. Consequently, plants conditioned with bioinputs allocated metabolic energy toward physiological defense rather than increasing yield accumulation or vegetative growth (Figure 6 and Figure 7). Similar growth limitations under restricted irrigation have been documented, where *B. aryabhattai* or soil amendments could not fully overcome drought-induced reductions in plant height, leaf area, and biomass in *Zea mays* [52] and *Mimosa caesalpiniifolia* [53]. Furthermore, high concentrations of potassium humate (T3 and T7) did not yield superior growth performance, which aligns with observations by Benito et al. [23] in *Arabidopsis thaliana*, where high soil humate applications exhibited mild phytotoxicity or growth-suppressive effects due to localized salt/osmotic imbalances.

Despite the growth restrictions during the vegetative stage, the investment in physiological homeostasis paid off during the reproductive phase. Inoculation with *B. aryabhattai* alone (T4) or combined with potassium humate (T1, T5) significantly increased the number of pods per plant (NP) by 22% to 51% under drought compared to unconditioned controls (Figure 7B). Additionally, combinations such as T2 (15 mg kg^−1^ soil KH) and T6 (15 mg kg^−1^ soil KH + 4 mL BA) enhanced 100-grain weight (HGW) under water deficit (Figure 7C). This protective effect on grain yield per plant components aligns with findings from Oliveira et al. [33] in common bean and Fuga et al. [30] in maize, where *inoculation with B. aryabhattai* mitigated yield losses under drought conditions. By maintaining cellular hydration, reducing oxidative damage to membranes, and preventing flower and pod abortion, the joint application of *B. aryabhattai* and potassium humate proved an effective biotechnological strategy for safeguarding cowpea yield components in water-limited environments, even when full vegetative growth is restricted [25,30,32].

Although this study provides important insights into the combined role of potassium humate and *B. aryabhattai* in mitigating drought stress in cowpea, certain limitations inherent to controlled trials must be acknowledged. The evaluations were conducted under greenhouse conditions in pot trials, which constrain spatial root expansion dynamics and water-foraging capacity compared to open-field soils. Furthermore, microclimatic variables in greenhouses (such as air temperature and relative humidity) are moderated. In contrast, field-grown crops face harsher, highly dynamic stress drivers, including high solar irradiance, wind, and elevated vapor pressure deficit. Another factor to consider is soil heterogeneity and competition from complex native soil microbial communities in open fields, which could influence the establishment, rhizosphere persistence, and bioactivity of the inoculated *B. aryabhattai* strain. Finally, assessments were focused on a single commercial cultivar (‘BRS Verdejante’), highlighting the need to validate these bioinput interactions across genotypes with contrasting drought tolerance. Therefore, future research should evaluate these formulations under open-field conditions across multiple seasons and soil types, and investigate molecular pathways and drought-responsive transcription factors to fully unravel the regulatory network driven by the co-application of humic substances and plant growth-promoting rhizobacteria.

## 4. Material and Methods

### 4.1. Experimental Site Characterization

**Figure 8:**
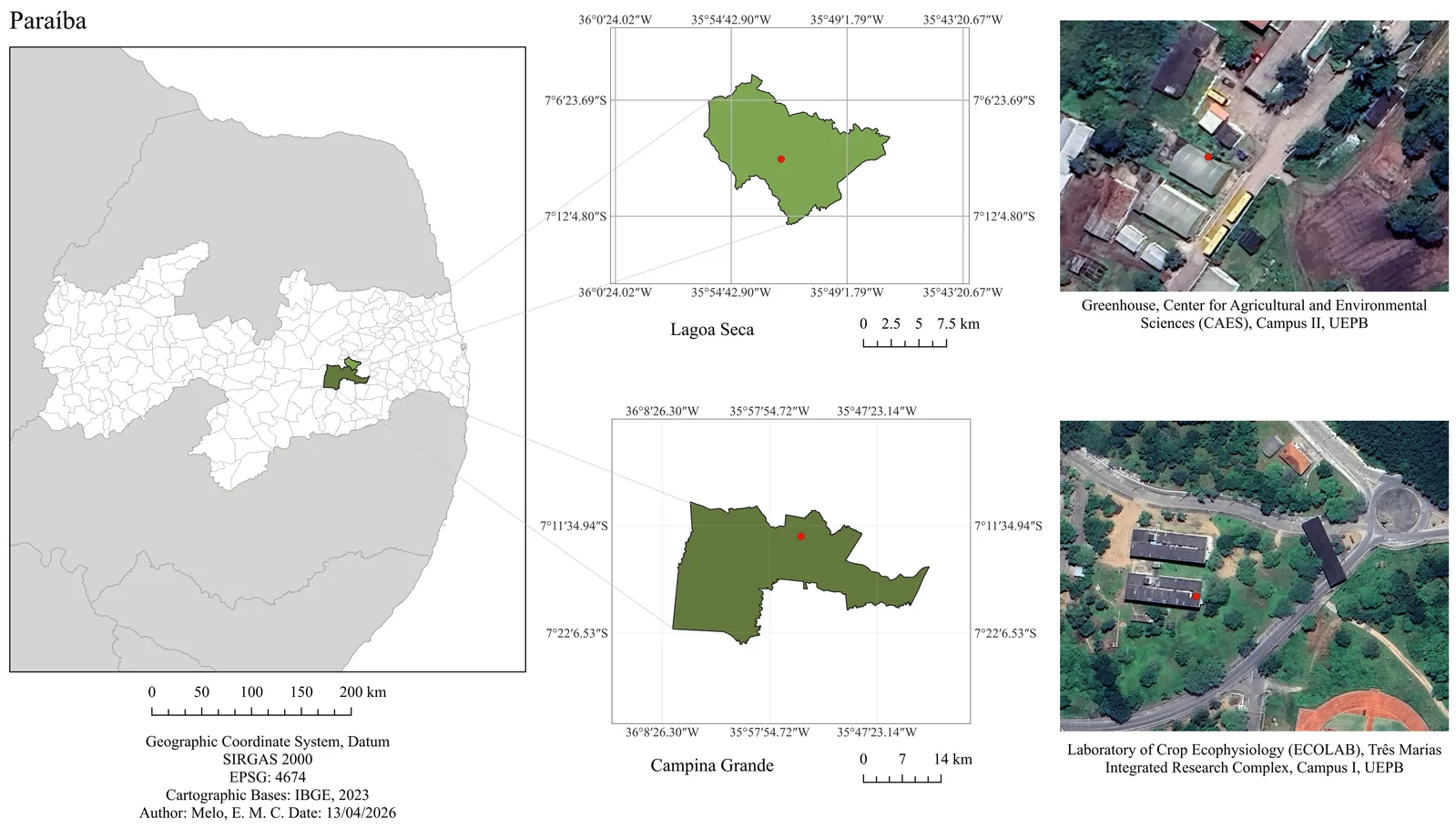
Location map of the experimental study area, highlighting the municipalities of Lagoa Seca and Campina Grande, Paraíba State, Brazil, along with details of the greenhouse at CCAA/UEPB and the ECOLAB laboratory at the Três Marias Complex, UEPB.

The experiment was conducted between August and October 2025 in a greenhouse located at the Center for Agricultural and Environmental Sciences (CCAA), *Campus* II of the State University of Paraíba (UEPB), Lagoa Seca, Paraíba, Brazil (latitude 7°09′22.6″ S, Longitude 35°52′8.46″ W, Altitude 634 m). Biochemical analyses were carried out at the Laboratory of Crop Ecophysiology (ECOLAB/UEPB), located in the Três Marias Integrated Research Complex (*Campus* I), Campina Grande, Paraíba, Brazil (07°13′50″ latitude, 35°52′52″ longitude, and 551 m altitude) (Figure 8).

**Figure 9:**
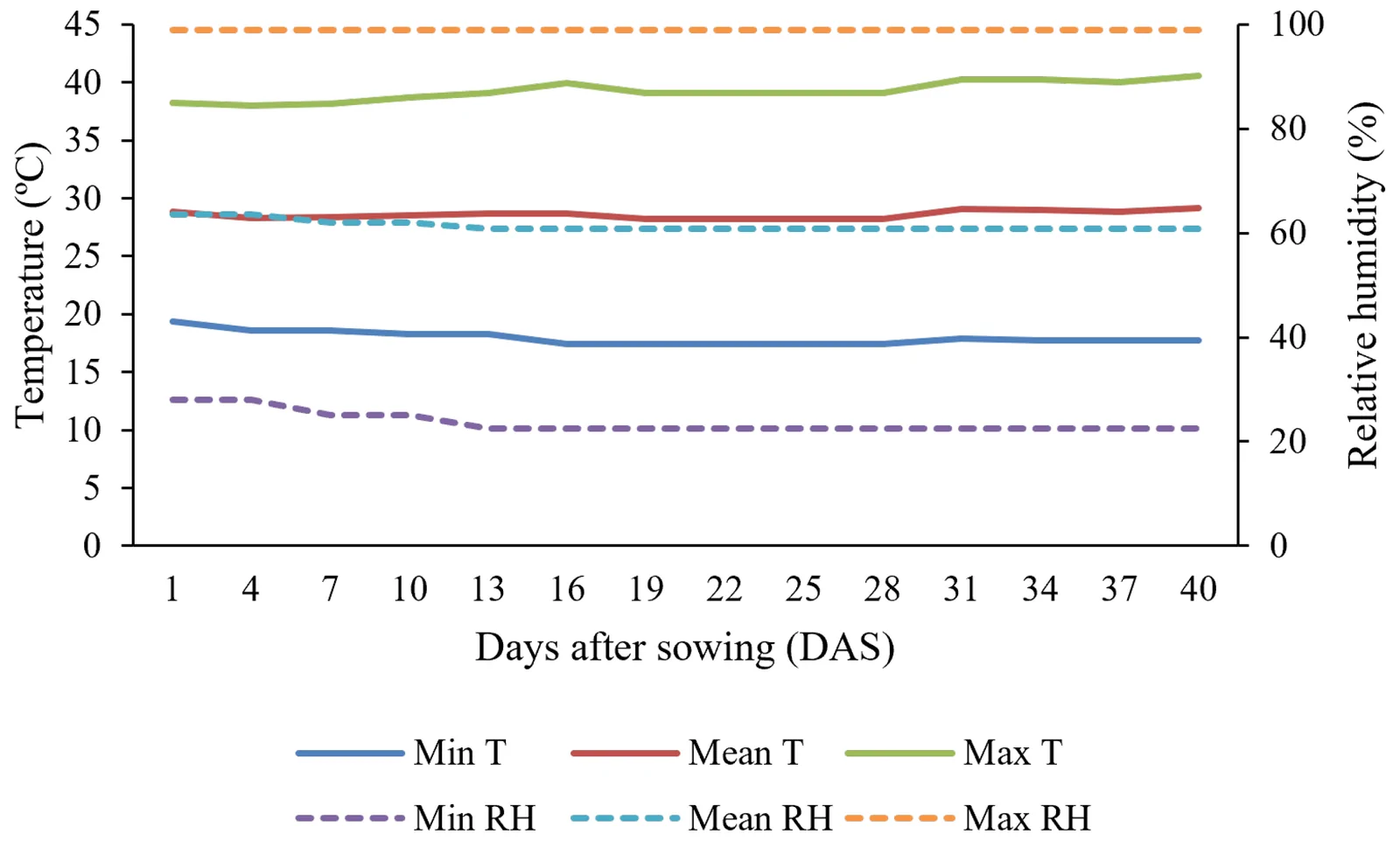
Maximum, minimum, and mean air temperatures and relative humidity recorded inside the greenhouse during the experimental period (August to October 2025).

Thermo-hygrometric conditions inside the greenhouse during the experimental period were monitored daily using a digital thermo-hygrometer (Exbom, FEPRO-MUT60OS) and are presented in Figure 9.

### 4.2. Experimental Design and Treatments

The experiment was arranged in a completely randomized design (CRD) in a 2 × 8 factorial layout with four replications, totaling 64 experimental units. The first factor consisted of two irrigation regimes: severe water restriction (W50—50% replacement of crop evapotranspiration, ETc) and full irrigation (W100—100% replacement of ETc). The second factor comprised eight biostimulant combinations derived from potassium humate (KH) and the commercial biological inoculant Auras^®^ (containing *B. aryabhattai* strain CMAA 1633 at a concentration of 1 × 10^8^ CFU mL^−1^). The KH doses (10, 15, and 20 mg kg^−1^ soil) were selected based on the effective soil conditioning ranges reported by Ullah et al. [51]. The inoculation rate of *B. aryabhattai* (BA) (4 mL kg^−1^ of seed, delivering ~1 × 10^8^ CFU mL^−1^ of seed) followed the technical recommendation for commercial seed treatment in leguminous crops and aligns with effective PGPR priming protocols established by Garcia Junior et al. [25] and Kavamura et al. [31] (Table 7).

### 4.3. Plant Material and Experimental Setup

Soil was collected from the 0–20 cm depth layer of an agricultural field at CCAA/UEPB, classified as a Regosol (Neossolo Regolítico according to the Brazilian Soil Classification System/Entisol according to USDA taxonomy). The soil was air-dried, sieved through a 2.0 mm mesh, homogenized, and analyzed for physical and chemical properties in accordance with standard methods [54]. The soil exhibited a sandy texture with the following baseline characteristics: 86.04% sand, 12.05% silt, and 1.91% clay; bulk density of 1.6 g cm^−3^; particle density of 2.7 g cm^−3^; total porosity of 39.8%; electrical conductivity (EC1:2.5) of 0.4 dS m^−1^; pH (in H_2_O, 1:2.5) of 6.5; organic matter of 0.9% (Walkley-Black); available phosphorus (Mehlich-1) of 14.5 mg dm^−3^; calcium of 1.9 cmol_c dm^−3^; magnesium of 1.5 cmol_c dm^−3^; sodium of 0.1 cmol_c dm^−3^; potassium of 0.3 cmol_c dm^−3^; sulfur of 3.8 cmol_c dm^−3^; potential acidity (H + Al) of 1.1 cmol_c dm^−3^; exchangeable aluminum of 0.00 cmol_c dm^−3^; sum of bases (SB) of 3.8 cmol_c dm^−3^; cation exchange capacity (CEC) of 4.9 cmol_c dm^−3^; and base saturation (V) of 77.9%. The trial was conducted in 25 L polyethylene pots lined at the bottom with non-woven fabric (TNT) to prevent soil loss during drainage, and the pots were filled with 30 kg of dry soil.

Cowpea seeds (*Vigna unguiculata* cv. ‘BRS Verdejante’) were obtained from the germplasm bank of Embrapa Meio-Norte. Seeds were visually selected to eliminate physical defects or malformations, surface-sterilized with 1% sodium hypochlorite for 3 min, rinsed with distilled water for 3 min, and air-dried for 48 h in a ventilated area [55].

Potassium humate (KH) was applied to the soil 72 h before sowing by manual homogenization using a hand cultivator. Seed inoculation with *B. aryabhattai* was performed by preparing a slurry containing 4 mL of Auras^®^ diluted in 4 mL of distilled water (8 mL total per kg of seed) and mixing thoroughly according to manufacturer guidelines. All seeds were treated with the fungicide Orthocide 500^®^ before sowing. Sowing was performed at a depth of 1 cm, with eight seeds per pot, and initial irrigation brought the soil to field capacity. Eight days after emergence (DAE), thinning was performed to leave four uniform plants per pot.

### 4.4. Irrigation Management

Irrigation regimes were managed using the gravimetric method described by Silva et al. [56]. Soil-filled pots were initially saturated with 3.5 L of water and covered with plastic bags to prevent evapotranspiration. After 24 h of free drainage, pot masses at field capacity (SMFC) were recorded. Pots were weighed every 48 h to determine soil mass after evapotranspiration (SMAE). The required water volume (RVW) for 100% ETc replacement was calculated using Equation (1):

(1) RVW=SMFC−SMAE

where: RVW = required water volume; SMFC = soil mass at field capacity; SMAE = soil mass after evapotranspiration.

The water volume for 50% ETc replacement (RVW50) was calculated using Equation (2):

(2) $$RVW\left(50\%\right)=\frac{RVW*50}{100}$$

where: RVW (50%) = 50% of the required water volume.

Water deficit was imposed abruptly 10 days after sowing (DAS, stage V3) by reducing the daily water replacement volume from 100% ETc to 50% ETc in the W50 treatment pots. The rationale for choosing an abrupt imposition of water restriction was to simulate the sudden onset of mid-season dry spells (‘veranicos’), which frequently interrupt the rainy season in semi-arid environments during early crop establishment. This sharp transition forces rapid cellular acclimation, allowing for the assessment of how potassium humate and *B. aryabhattai* prime immediate osmotic adjustment and antioxidant defenses under dehydration. Pot weighings were performed in the morning using a digital platform scale with 200 kg capacity and 1 g precision (Electro, KG-200). Over the course of the experiment, a total of 1390 L of water was applied (928 L for W100 plants and 464 L for W50 plants).

### 4.5. Variables Analyzed

Biochemical, physiological, and water *status* evaluations were performed using the third fully expanded trifoliate leaf collected at 36 days after sowing (DAS, stage R1).

#### 4.5.1. Water *Status* Indicators and Membrane Damage

Relative water content (RWC) was determined as described by Brito et al. [57] and Ferraz et al. [58]. Five fresh leaf discs (5 mm diameter) per replicate were cut, weighed to obtain fresh mass (FMD), and immersed in hermetically sealed aluminum capsules containing 10 mL of distilled water for 24 h at room temperature. Discs were then blotted dry and weighed to determine turgid mass (TMD). Finally, discs were oven-dried at 60 °C for 48 h to obtain dry mass (DMD). RWC was calculated using Equation (3):

(3) $$RWC=\frac{FMD-DMD}{TMD-DMD}*100$$

where: RWC = relative water content; FMD = fresh mass of discs; DMD = dry mass of discs; TMD = turgid mass of discs.

Electrolyte leakage (EL) was evaluated as described by Brito et al. [57]. Five leaf discs (5 mm) per experimental unit were washed, placed in test tubes with 10 mL of deionized water, and incubated at room temperature for 24 h. Initial electrical conductivity (iEC) was measured using a portable conductivity meter (Waterproof, YH-1387). Tubes were then incubated in a water bath at 100 °C for 60 min, cooled to room temperature, and final electrical conductivity (fEC) was measured. EL was calculated using Equation (4):

(4) $$EL=\frac{iEC}{fEC}\;*100$$

where: EL = electrolyte leakage; iEC = initial electrical conductivity; fEC = final electrical conductivity.

Lipid peroxidation was quantified by malondialdehyde (MDA) content, according to Heath and Packer [59]. Fresh leaf tissue (0.2 g) was homogenized in 1.5 mL of 6% (*w*/*v*) trichloroacetic acid (TCA) under refrigerated conditions. The homogenate was centrifuged at 12,000× *g* for 15 min at 4 °C. A 500 µL aliquot of the supernatant was reacted with 2 mL of 20% TCA containing 0.5% (*w*/*v*) thiobarbituric acid (TBA). The mixture was heated at 95 °C for 60 min, rapidly cooled in an ice bath, and absorbance was measured at 532 nm and 600 nm (for non-specific turbidity correction). Results were expressed as nmol MDA g^−1^ fresh mass (FM).

#### 4.5.2. Antioxidant Defense System Activity

To determine the activity of antioxidant enzymes (superoxide dismutase, catalase, and ascorbate peroxidase) and total soluble proteins, 200 mg of fresh material was homogenized with 2 mL of 50 mM phosphate buffer (pH 7.0), supplemented with ascorbic acid (0.1 mM), EDTA (0.1 mM), and polyvinylpyrrolidone (5%). Subsequently, the extracts were centrifuged at 20,000× *g* for 20 min at 4 °C. Following this process, the supernatant was transferred to 2.5 mL plastic tubes (Eppendorf^®^) and stored at −20 °C for subsequent analyses.

SOD activity was determined according to the methodology of Beauchamp and Fridovich [60], based on the enzyme’s ability to inhibit the photoreduction of nitroblue tetrazolium (NBT) in the plant extract. Absorbance readings of the solutions containing the respective enzyme extracts were performed using a spectrophotometer set at 560 nm, and activity was expressed in UA g^−1^ FM.

CAT activity was quantified as described by Sudhakar et al. [61], based on the consumption of hydrogen peroxide (H_2_O_2_) by the extract. The reaction was initiated by adding the enzyme extract (150 μL) to a quartz cuvette containing 2.85 mL of the reaction mixture, which consisted of 1950 μL of potassium phosphate buffer (100 mM, pH 7.5), 150 μL of extraction buffer (0.1 mM ascorbic acid, 0.1 mM EDTA, and 3% polyvinylpyrrolidone), and 750 μL of hydrogen peroxide (H_2_O_2_) solution (50 mM). Absorbance readings were taken over two minutes at 10 s intervals using a spectrophotometer set to 240 nm. Activity was expressed in µM H_2_O_2_ min^−1^ g^−1^ FM.

APX activity was determined according to the method of Nakano and Asada [62]. A 100 μL aliquot of the extract was mixed with 2.7 mL of reaction medium consisting of 50 mM potassium phosphate buffer (pH 6.0) supplemented with 0.8 mM ascorbic acid. The reaction was initiated by adding 200 μL of hydrogen peroxide (H_2_O_2_, 2 mM), and the decrease in absorbance at 290 nm was monitored for 1 min, with readings taken every 10 s. Calculations were performed based on the Beer–Lambert law, and final APX activity was expressed in µM ascorbate min^−1^ g^−1^ FM.

#### 4.5.3. Photosynthetic Pigments

Photosynthetic pigments, chlorophyll *a* (Chl *a*), chlorophyll *b* (Chl *b*), total chlorophyll (Chl *t*), total carotenoids (CAR), and total anthocyanins (ANT), were extracted from 200 mg fresh leaf samples in 3 mL of cold 80% (*v*/*v*) acetone/Tris-HCl buffer (pH 7.8) under low light according to Sims and Gamon [63]. Absorbance was read at 663, 647, 470, and 537 nm. Pigment concentrations were calculated using Equations (5)–(9) and expressed in mg 100 g^−1^ FM:

(5) $$Chl\;a=\{\left[\left(0.01373*A663\right)-\left(0.000897*A537\right)\right]-\;(0.003046*A647)\}$$

(6) $$Chl\;b=\{\left[\left(0.02405*A647\right)-\left(0.004305*A537\right)\right]-\left(0.005507*A663\right)\}$$

(7) Chl t=Chl a+Chl b

(8) $$CAR=\frac{\left\{A470-\left[17.1*\left(Chl\;a+Chl\;b\right)\right]\right\}-\left(9.479*ANT\right)}{119.26}$$

(9) $$ANT=\{\left[\left(0.08173*A537\right)-\left(0.00697*A647\right)\right]-\left(0.002228*A663\right)\}\;$$

#### 4.5.4. Osmotic Adjustment Mechanisms

Total soluble protein (TSP) quantification was performed according to Bradford [64]; 22.7 μL of enzyme extract was added to test tubes, followed by 700 μL of Bradford reagent. The mixture was gently agitated and kept at room temperature for 15 min before being read in a spectrophotometer set to 595 nm. TSP activity was expressed in mg TSP g^−1^ FM.

Free proline quantification was performed using the colorimetric method proposed by Bates et al. [65] and modified by Bezerra Neto and Barreto [66]. Fresh leaf tissue (250 mg) was macerated in 5 mL of 3% (*v*/*v*) sulfosalicylic acid and centrifuged at 2000 rpm for 10 min; the supernatant was then aspirated and stored in 2.5 mL centrifuge tubes for subsequent determination of proline concentration. The supernatant was read in a spectrophotometer at 520 nm, using pure toluene as a blank to zero the instrument. Free proline concentration was quantified based on an L-proline standard curve (0, 5, 10, 20, 25, and 50 mg L^−1^) and expressed in µmol proline g^−1^ FM.

Total soluble sugar (TSS) content was determined using the anthrone colorimetric method described by Yemm and Willis [67]. For the assay, 50 μL sample aliquots were added to test tubes, followed by 1950 μL of distilled water and 2000 μL of anthrone reagent. After addition, the tubes were agitated again to homogenize the reaction mixture. Subsequently, the tubes were placed in a water bath at 100 °C for 3 min and then in an ice bath for spectrophotometric reading at 620 nm. TSS content was quantified against a glucose standard curve, and results were expressed in mg TSS g^−1^ FM.

#### 4.5.5. Plant Growth

Plant height (PH) and stem diameter (SD) were measured on one plant per pot at the R1 stage (36 days after sowing). PH was measured from the base of the stem to the apical meristem using a graduated ruler (cm), and SD was recorded at the stem base using a digital caliper (MTX, 316119; mm). Shoot dry mass (SDM) was evaluated by harvesting the shoot, drying it in a forced-air circulation oven at 70 °C for 72 h (until a constant mass was achieved), and weighing it on an analytical balance (±0.0001 g precision; Shimadzu, ATY224R). Total leaf area (TLA) was determined using a benchtop leaf area meter (LI-COR, LI-3100C) and expressed in mm^2^.

#### 4.5.6. Agronomic Yield Components

Yield components were evaluated at harvest (R5 stage, 55 DAS). The parameters included: number of pods per plant (NP, pods plant^−1^), 100-grain mass (HGW, g), and total grain yield per plant (GY, g per plant). Grains were weighed on an analytical balance (±0.0001 g; Shimadzu, ATY224R).

### 4.6. Statistical Analysis

Data were tested for normality using the Shapiro–Wilk test (*p* ≥ 0.05) [68]. Once the normality assumptions were met, the data were subjected to a two-way analysis of variance (ANOVA; F-test; *p* ≤ 0.05). Means for irrigation regimes were compared using Student’s *t*-test (*p* ≤ 0.05), and biostimulant treatment means were compared using the Scott–Knott cluster test (*p* ≤ 0.05). All statistical analyses were performed using SISVAR software version 5.6 [69].

## 5. Conclusions

Water stress compromised cell membrane integrity and reduced photosynthetic pigment levels in *Vigna unguiculata* (L.) Walp. cv ‘BRS Verdejante’, triggering adaptive responses involving osmoregulation and the activation of the enzymatic antioxidant system. The single application of *Bacillus aryabhattai* and low-to-moderate doses of potassium humate, as well as their co-application (10 mg kg^−1^ soil KH + 4 mL kg^−1^ seed BA), effectively preserved leaf water *status*, protected cell membrane integrity, and stabilized photosynthetic pigments in cowpea (cv. ‘BRS Verdejante’) under severe water deficit (50% ETc). From a practical management perspective, this low-dose bioinput co-application offers a promising strategy to support crop management during severe water restriction by mitigating oxidative damage and safeguarding pod formation. Nevertheless, open-field validation across contrasting genotypes, soil types, and climate conditions remains essential to confirm the validity of these physiological responses and support agronomic recommendations for commercial production systems.

## Figures and Tables

**Table 1:** Summary of the analysis of variance for the variables relative water content (RWC, %), electrolyte leakage (EL, %) and lipid peroxidation (MDA, nmol MDA g^−1^ FM) of cowpea plants (*Vigna unguiculata* cv. ‘BRS Verdejante’) subjected to eight different combinations of potassium humate (KH) and *Bacillus aryabhattai* (BA) under two irrigation regimes.

F-Test (Pr > Fc)				
SV	Sum of Squares			
	DF	RWC	EL	MDA
IR	1	0.395 **	3.942 **	3.062 **
COM	7	0.381 **	2.579 **	13.355 **
IR × COM	7	0.053 *	2.358 **	0.088 **
Residue	48	0.021	0.016	0.088
Average		9.65	5.23	12.47
CV (%)		1.69	2.47	2.39

** Significant at 1% (*p* ≤ 0.01); * significant at 5% (*p* ≤ 0.05). SV—source variation. IR—corresponds to the two irrigation regimes (W50—50% and W100—100% of ETc); COM—refers to the eight biostimulant combinations derived from potassium humate and the biological commercial inoculant Auras^®^ used in the experiment (control—without biostimulant application; T1—10 mg kg^−1^ soil KH; T2—15 mg kg^−1^ soil KH; T3—20 mg kg^−1^ soil KH; T4—4 mL kg^−1^ seeds BA; T5—10 mg kg^−1^ soil KH + 4 mL kg^−1^ seeds BA; T6—15 mg kg^−1^ soil KH + 4 mL kg^−1^ seeds BA; T7—20 mg kg^−1^ soil KH + 4 mL kg^−1^ seeds BA); CV—coefficient of variation.

**Table 2:** Summary of the analysis of variance for the variables chlorophyll *a* (Chl *a*, mg 100 g^−1^ FM), chlorophyll *b* (Chl *b*, mg 100 g^−1^ FM), total chlorophyll (Chl *t*, mg 100 g^−1^ FM), total carotenoids (CAR, mg 100 g^−1^ FM), and total anthocyanins (ANT, mg 100 g^−1^ FM) of cowpea plants (*Vigna unguiculata* cv. ‘BRS Verdejante’) subjected to eight different combinations of potassium humate (KH) and *Bacillus aryabhattai* (BA) under two irrigation regimes.

F-Test (Pr > Fc)						
SV	Sum of Squares					
	DF	Chl *a*	Chl *b*	Chl *t*	CAR	ANT
IR	1	0.0003 ^NS^	0.000036 ^NS^	0.0001 ^NS^	0.0007 **	1.91 × 10^−8 NS^
COM	7	0.001 **	0.000068 **	0.00026 **	0.0006 **	0.002 **
IR × COM	7	0.0003 *	0.000097 **	0.00027 **	0.0003 **	0.0009 *
Residue	48	0.00032	0.00002	0.000048	0.000045	0.000062
Average		0.16	0.01	0.04	0.11	0.08
CV (%)		10.94	23.07	14.65	5.83	9.37

** Significant at 1% (*p* ≤ 0.01); * significant at 5% (*p* ≤ 0.05); NS = not significant. SV—source variation. IR—corresponds to the two irrigation regimes (W50—50% and W100—100% of ETc); COM—refers to the eight biostimulant combinations derived from potassium humate and the biological commercial inoculant Auras^®^ used in the experiment (control—without biostimulant application; T1—10 mg kg^−1^ soil KH; T2—15 mg kg^−1^ soil KH; T3—20 mg kg^−1^ soil KH; T4—4 mL kg^−1^ seeds BA; T5—10 mg kg^−1^ soil KH + 4 mL kg^−1^ seeds BA; T6—15 mg kg^−1^ soil KH + 4 mL kg^−1^ seeds BA; T7—20 mg kg^−1^ soil KH + 4 mL kg^−1^ seeds BA); CV—coefficient of variation.

**Table 3:** Summary of the analysis of variance for the variables superoxide dismutase (SOD, UA g^−1^ FM), catalase (CAT, µmol H_2_O_2_ min^−1^ g^−1^ FM), and ascorbate peroxidase (APX, mM min^−1^ g^−1^ FM) of cowpea plants (*Vigna unguiculata* cv. ‘BRS Verdejante’) subjected to eight different combinations of potassium humate (KH) and *Bacillus aryabhattai* (BA) under two irrigation regimes.

F-Test (Pr > Fc)				
SV	Sum of Squares			
	DF	SOD	CAT	APX
IR	1	3.502 **	1.940 **	0.080 **
COM	7	6.975 **	4.490 **	0.131 **
IR × COM	7	0.975 **	1.522 **	0.052 **
Residue	48	0.072	0.038	0.009
Average		6.77	5.81	1.18
CV (%)		3.97	3.35	8.01

** Significant at 1% (*p* ≤ 0.01). SV—source variation. IR—corresponds to the two irrigation regimes (W50—50% and W100—100% of ETc); COM—refers to the eight biostimulant combinations derived from potassium humate and the biological commercial inoculant Auras^®^ used in the experiment (control—without biostimulant application; T1—10 mg kg^−1^ soil KH; T2—15 mg kg^−1^ soil KH; T3—20 mg kg^−1^ soil KH; T4—4 mL kg^−1^ seeds BA; T5—10 mg kg^−1^ soil KH + 4 mL kg^−1^ seeds BA; T6—15 mg kg^−1^ soil KH + 4 mL kg^−1^ seeds BA; T7—20 mg kg^−1^ soil KH + 4 mL kg^−1^ seeds BA); CV—coefficient of variation.

**Table 4:** Summary of the analysis of variance for the variables free proline (PRO, µmol g^−1^ FM), total soluble sugars (TSS, mg TSS g^−1^ FM) and total soluble proteins (TSP, mg TSP g^−1^ FM) of cowpea plants (*Vigna unguiculata* cv. ‘BRS Verdejante’) subjected to eight different combinations of potassium humate (KH) and *Bacillus aryabhattai* (BA) under two irrigation regimes.

F-Test (Pr > Fc)				
SV	Sum of Squares			
	DF	PRO	TSS	TSP
IR	1	0.003 **	11.94 **	0.05 **
COM	7	0.001 **	4.71 **	0.03 **
IR × COM	7	0.0001 *	0.4 ^NS^	0.01 **
Residue	48	0.00005	0.607	0.004
Average		0.04	6.94	0.88
CV (%)		15.26	11.23	7.27

** Significant at 1% (*p* ≤ 0.01); * significant at 5% (*p* ≤ 0.05); NS = not significant. SV—source variation. IR—corresponds to the two irrigation regimes (W50—50% and W100—100% of ETc); COM—refers to the eight biostimulant combinations derived from potassium humate and the biological commercial inoculant Auras^®^ used in the experiment (control—without biostimulant application; T1—10 mg kg^−1^ soil KH; T2—15 mg kg^−1^ soil KH; T3—20 mg kg^−1^ soil KH; T4—4 mL kg^−1^ seeds BA; T5—10 mg kg^−1^ soil KH + 4 mL kg^−1^ seeds BA; T6—15 mg kg^−1^ soil KH + 4 mL kg^−1^ seeds BA; T7—20 mg kg^−1^ soil KH + 4 mL kg^−1^ seeds BA); CV—coefficient of variation.

**Table 5:** Summary of the analysis of variance for the variables plant height (PH, cm), stem diameter (SD, mm), total leaf area (TLA, mm^2^) and shoot dry mass (SDM, g) of cowpea plants (*Vigna unguiculata* cv. ‘BRS Verdejante’) subjected to eight different combinations of potassium humate (KH) and *Bacillus aryabhattai* (BA) under two irrigation regimes.

F-Test (Pr > Fc)					
SV	Sum of Squares				
	DF	PH	SD	TLA	SDM
IR	1	7.96 **	0.12 **	232,183.03 **	8.70 **
COM	7	0.81 **	0.01 ^NS^	11,965.69 **	0.49 ^NS^
IR × COM	7	0.68 **	0.009 ^NS^	6196.79 **	0.38 ^NS^
Residue	48	0.019	0.013	1928.05	0.453
Average		5.10	2.50	528.71	3.57
CV (%)		2.76	4.58	7.54	18.83

** Significant at 1% (*p* ≤ 0.01); NS = not significant. SV—source variation. IR—corresponds to the two irrigation regimes (W50—50% and W100—100% of ETc); COM—refers to the eight biostimulant combinations derived from potassium humate and the biological commercial inoculant Auras^®^ used in the experiment (control—without biostimulant application; T1—10 mg kg^−1^ soil KH; T2—15 mg kg^−1^ soil KH; T3—20 mg kg^−1^ soil KH; T4—4 mL kg^−1^ seeds BA; T5—10 mg kg^−1^ soil KH + 4 mL kg^−1^ seeds BA; T6—15 mg kg^−1^ soil KH + 4 mL kg^−1^ seeds BA; T7—20 mg kg^−1^ soil KH + 4 mL kg^−1^ seeds BA); CV—coefficient of variation.

**Table 6:** Summary of the analysis of variance for the variables grain yield per plant (GY, g), pods per plant (NP, pods plant^−1^) and 100-grain weight (HGW, g) of cowpea plants (*Vigna unguiculata* cv. ‘BRS Verdejante’) subjected to eight different combinations of potassium humate (KH) and *Bacillus aryabhattai* (BA) under two irrigation regimes.

F-Test (Pr > Fc)				
SV	Sum of Squares			
	DF	GY	NP	HGW
IR	1	4609.03 **	1097.26 **	0.11 ^NS^
COM	7	9.558 ^NS^	5.79 **	5.30 **
IR × COM	7	9.996 *	4.51 *	7.20 **
Residue	48	4.56	1.89	0.722
Average		13.67	17.07	21.20
CV (%)		10.06	4.98	10.07

** Significant at 1% (*p* ≤ 0.01); * significant at 5% (*p* ≤ 0.05); NS = not significant. SV—source variation. IR—corresponds to the two irrigation regimes (W50—50% and W100—100% of ETc); COM—refers to the eight biostimulant combination derived from potassium humate and the biological commercial inoculant Auras^®^ used in the experiment (control—without biostimulant application; T1—10 mg kg^−1^ soil KH; T2—15 mg kg^−1^ soil KH; T3—20 mg kg^−1^ soil KH; T4—4 mL kg^−1^ seeds BA; T5—10 mg kg^−1^ soil KH + 4 mL kg^−1^ seeds BA; T6—15 mg kg^−1^ soil KH + 4 mL kg^−1^ seeds BA; T7—20 mg kg^−1^ soil KH + 4 mL kg^−1^ seeds BA); CV—coefficient of variation.

**Table 7:** Experimental treatments resulting from combinations of potassium humate (KH) and *Bacillus aryabhattai* (BA).

Treatments	Combinations Description
Control	0 mg kg^−1^ soil KH + 0 mL kg^−1^ of seeds BA
1	10 mg kg^−1^ soil KH + 0 mL kg^−1^ of seeds BA
2	15 mg kg^−1^ soil KH + 0 mL kg^−1^ of seeds BA
3	20 mg kg^−1^ soil KH + 0 mL kg^−1^ of seeds BA
4	0 mg kg^−1^ soil KH + 4 mL kg^−1^ of seeds BA
5	10 mg kg^−1^ soil KH + 4 mL kg^−1^ of seeds BA
6	15 mg kg^−1^ soil KH + 4 mL kg^−1^ of seeds BA
7	20 mg kg^−1^ soil KH + 4 mL kg^−1^ of seeds BA

## Data Availability

The datasets generated during and/or analyzed during the current study are available from the corresponding author on reasonable request.
